# Concerning Cat-Gut Infection

**Published:** 1890-02

**Authors:** 


### CONCERNING CAT-GUT INFECTION.

FROM PROCEEDINGS OF THE 62D MEETING OF GERMAN NATURALISTS AND PHYSICIANS AT HEIDELBERG.

Translation from Centralblatt für Chirurgie.

C. Brunner (Zurich). The speaker took the task of answering, from clinical experience and bacteriological experiment, the question: "Are the customary methods of disinfection capable of presenting completely aseptic cat-gut?" The knowledge concerning infection with cat-gut, gathered from a review of the literature and from communications by letter, embraces observations by Zweiffel, v. Volkmann, Kocher, Neuber, Socin, v. Mosetig, Kappeler and Haffter. While, however, all the others spoke of infection with carbolized cat-gut, Kocher alone mentioned "juniper" and "sublimate" cat-gut as causing serious hindrance of asepsis. In the experimental part of his work the speaker had sought the "germ-contents" of the commercially prepared cat-gut. Thus it was shown that "sublimate-gut" and that treated with dry heat, after Reverdin's method, was completely sterile; that on the other hand, carbolic acid, chromic acid and juniper cat-gut presented numerous colonies of organisms, among these, also, a non-pathogenic bacillus, which the observer could not identify with any previously known variety. Further, the speaker had tried the absorptive capacity of gut for the various disinfecting agents, allowing them to work into material taken from puppies having splenic disease. The experiments on "raw" cat-gut, giving cultivations of limited colonies of splenic-disease-germs, gave the following results, viz.:

(1) Six hours' action of bichloride, 1—1000, gave absolute freedom from germs in like cultures.

(2) After two hours' action of dry heat, 140°C(?), all cultures proved sterile.

(3) After thirty-six hours' action of oil of juniper, the cat-gut showed in the majority of the cultures numerous "colonies."

(4) Preservation in 20 per cent. carbolized oil gave, to all tests, freedom from germs.

In the discussion Morian (of Essen) warned against considering sterility proven if no germs appeared after trial in cultivation material. To his view the animal presented a more delicate reagent. Mice and puppies may die from infected—but apparently disinfected—cat-gut, which presented no germs in artificial cultivations.

